# Relationships Between Anxiety, Perceived Vulnerability to Disease, and Smartphone Use During Coronavirus Disease 2019 Pandemic in a Sample of Italian College Students

**DOI:** 10.3389/fpsyg.2021.692503

**Published:** 2021-07-14

**Authors:** Concetta De Pasquale, Maria Luisa Pistorio, Federica Sciacca, Zira Hichy

**Affiliations:** ^1^Department of Educational Sciences, University of Catania, Catania, Italy; ^2^Department of General Surgery and Medical-Surgical Specialties, University Hospital of Catania, Catania, Italy

**Keywords:** anxiety, perceived vulnerability, smartphone addiction, college students, fear COVID-19

## Abstract

**Introduction:** As of March 2020, coronavirus disease 2019 (COVID-19) has been declared a “pandemic” by the WHO. This has led to the need for governments around the world to implement restrictive containment and isolation measures to stem the spread of the virus; these measures have included social distancing, isolation, and quarantine. The fear of contagion has been indicated as one of the causes of stress, anxiety, depression, and insomnia in the general population. With respect to the response of young people to the pandemic, the category of University students deserves further attention. The sudden change in “University” habits (i.e., poor interaction with teachers and colleagues, disturbing learning environment, and difficulty in adapting to online learning), the consequent loss of a social network, and the economic problems in their families have seriously affected the psychophysical well-being of University students. The aim of this study was to explore, in a sample of Italian University students, the relationships among anxiety, perceived vulnerability to disease, and smartphone use during the COVID-19 pandemic.

**Methods:** A sample of 194 volunteer college students (i.e., 86 males and 108 females) aged between 18 and 30 years (*M* = 21.74; *SD* = 2.39) were recruited to participate in this study. Participants were recruited through an online questionnaire sent to students of the University of Catania, Italy, and distributed from September 2020 to January 2021. The volunteer participants were given an online protocol that included the Fear of COVID-19 Scale (FCV-19S) for the evaluation of fear, the Perceived Vulnerability to Disease (PVD) for the evaluation of perceived vulnerability to disease, the State-Trait Anxiety Inventory (STAI) for the evaluation of trait and state anxiety, and the Smartphone Addiction Scale Short Version for Adolescents and Young Adults (SAS-SV) for the evaluation of use, abuse, or addiction of smartphone use.

**Results:** The fear of COVID-19 did not reach an intensity such as to be defined as serious (i.e., fear score: 15.53) in the whole sample. Both men and women showed a high risk of smartphone addiction (i.e., score of males: 28.33 and score of females: 26.88) in SAS-SV. University students showed moderate trait and state anxiety [i.e., a score of 51.60 in Trait Anxiety Inventory (TAI) and a score of 47.21 in State Anxiety Inventory (SAI)] in STAI. In addition, students showed moderate perceived vulnerability to disease (i.e., a score of 51.51) in PVD. The results showed that fear of COVID-19 and trait anxiety appear to be the predictors of SAI and PVD but not the predictors of risk of smartphone addiction (SAS-SV).

**Conclusions:** The data highlighted the presence of a perception of vulnerability to infections in subjects in which there was also a moderate anxiety, both state and trait, associated with the fear of the COVID-19 pandemic. It is hoped that a large part of the population will soon be vaccinated, including University students, and therefore, it would be desirable to carry out further assessments in the post-vaccine phase to highlight any differences in the state of anxiety and the perception of vulnerability to infections. The possible positive role of the use of smartphones in maintaining social contacts should also be emphasized.

## Introduction

Coronavirus disease 2019 (COVID-19) has rapidly expanded in Europe, North America, Asia, and the Middle East. Based on the alarming levels of prevalence and severity, the Director-General of the WHO on March 11, 2020 called the COVID-19 situation a pandemic (Bedford et al., 2020). To respond to this pandemic, many countries have taken containment measures to reduce the demand for hospitalization and protect those most vulnerable to infection (De Coninck et al., 2020). To stop the spread of the diseases, a vaccine is needed. But, in the absence of the vaccine, people should maintain social distancing. To maintain social distancing, they should obey the modeling rule (Khajanchi and Sarkar, 2020; Samui et al., 2020; Sarkar et al., 2020).

The lack of immunity and vaccines against the virus, its exponential spread, and high mortality rate have contributed to the perception of a largely uncontrollable and unpredictable threat, causing emotional distress up to full-blown psychopathology (Asmundson et al., 2020; Fiorillo et al., 2020).

In this context, an important factor is the impact of the effect of media. The COVID-19 dynamics have changed due to the incorporation of media-related awareness such as the use of face masks, non-pharmaceutical interventions, and hand sanitization (Rai et al., 2021).

Is there any experimental data to validate the mathematical model? Subhas et al. (2021) described the basic reproduction number R_0 and its impact on the COVID-19 pandemic in India. The basic reproduction number R_0 is one of the most crucial quantities in infectious diseases, as R_0 measures how contagious a disease is. For R_0 <1, the disease is expected to stop spreading, but for R_0 = 1, an infected individual can infect on an average of 1 person, that is, the spread of the disease is stable. The disease can spread and become epidemic if R_0 must be >1.

Overall, the importance of possible negative effects of COVID-19 at the emotional level on the population has been documented by several surveys.

The study carried out in China found moderate-to-severe anxiety in about one-third of the sample examined, a high prevalence of sleep disorders, and generalized symptoms of anxiety disorder, especially in young people and health professionals (Huang and Ning, 2020; Wang et al., 2020a).

A study carried out on the Italian population showed a high rate of post-traumatic stress symptoms, depression, anxiety, insomnia, and adaptation disorder symptoms, with higher probabilities in young women (Rossi et al., 2020).

Furthermore, several studies have shown that the COVID-19 infection in association with infodemia increased the risk of psychiatric/psychological disorders by stimulating the hypothalamus–pituitary–adrenal (HPA) axis and has shown how dangerous this is for the public, especially for pregnant women and disabled people (Khan et al., 2020, 2021; Nabi et al., 2020).

In addition, Wang et al. (2020b) showed that the deprivation of social interaction, such as that occurred during the COVID-19 pandemic, can stimulate the HPA axis, resulting in an increased secretion of stress hormones and a decreased secretion of social hormones and causing the dysregulation of social behaviors in people.

Although a significant correlation has been shown between perceived vulnerability to disease (PVD) and emotional distress, individuals can cope better with the psychological and social threats of the pandemic if they can manage anxiety and depression, despite the high levels of fear of the pandemic infection (Jungmann and Witthöft, 2020; Li et al., 2021).

The study by Stangier et al. (2021) highlighted how the perceived vulnerability to infections predicted an increase in preventive behavior, while the aversion to germs predicted an increase in preventive behavior and a decrease in risky behavior.

With respect to the response of young people to the pandemic, the category of University students deserves further attention. The sudden change in “University” habits (i.e., poor interaction with teachers and colleagues, disturbing learning environment, and difficulty in adapting to online learning), the consequent loss of a social network, and the economic problems in their families have seriously affected the psychophysical well-being of University students (Stangier et al., 2021).

Huckins et al. (2020) studied the impact of COVID-19 on mental health and behavior in a sample of 217 American college students. The authors highlighted how the fear associated with the pandemic caused an increase in the symptoms of anxiety, depression, and some behaviors to watch out for such as continued smartphone use in the young people examined.

The study by Kleiman et al. (2020) was conducted on 140 college students in the United States and highlighted the increased desire to drink and use drugs in the most anxious and depressed subjects due to COVID-19.

In the study by Elhai et al. (2020) conducted on 908 subjects in China, it was found that COVID-19 anxiety was related to the severity of problematic smartphone use (PSU), depression, and anxiety; however, COVID-19 anxiety did not predict a greater severity of PSU. To what extent can a pandemic lead to risky behavior? However, the predictive value of the levels of anxiety and stress of PSU has been highlighted by the international literature even in the periods preceding the pandemic (De Pasquale et al., 2015, 2018, 2019; Vahedi and Saiphoo, 2018).

Based on these premises, the aim of this study was to explore, in a sample of Italian University students, the relationships between anxiety, perceived vulnerability to disease, and smartphone use during the COVID-19 pandemic.

## Methods

### Participants

A sample of 194 volunteer college students (i.e., 86 males and 108 females) aged between 18 and 30 years (*M* = 21.74; *SD* = 2.39) were recruited to participate in this study. Participants were recruited through an online questionnaire sent to students of the University of Catania, Italy, and distributed from September 2020 to January 2021. The survey was also shared on the Facebook page of the Department of Educational Sciences of the University of Catania. All participants provided online informed consent and answered the questionnaire anonymously.

### Measures

The volunteer participants were given an online protocol that included the Fear of COVID-19 Scale (FCV-19S) for the evaluation of fear, the Perceived Vulnerability to Disease (PVD) for the evaluation of perceived vulnerability to disease, the State-Trait Anxiety Inventory (STAI) for the evaluation of trait and state anxiety, and the Smartphone Addiction Scale Short Version for Adolescents and Young Adults (SAS-SV) for the evaluation of use, abuse, or addiction of smartphone use.

The FCV-19S (Ahorsu et al., 2020) is a questionnaire composed of 7 items (e.g., “I'm very afraid of coronavirus-19,” “It makes me uncomfortable to think about coronavirus-19,” and “I can't sleep because I worry about getting coronavirus-19”), with a 5-point Likert response scale (i.e., 1 = strongly disagree to 5 = strongly agree), which assesses the fear of COVID-19. The scale showed a reliability score of 0.874. The score ranges from 7 to 35, with higher scores correlated with higher fear.

The Perceived Vulnerability to Disease (PVD; Park and Justin, 2009) is a questionnaire for the evaluation of individual differences in perceived vulnerability of disease and emotional distress in the presence of potential transmission of diseases. It is composed of 15 items with a 7-point response scale (i.e., 1 = strongly disagree to 7 = strongly agree), divided into two subscales or factors. The first factor [i.e., Perceived Infectability (PI)] is composed of seven items to evaluate beliefs about immunological functioning and personal susceptibility to infectious diseases. The second factor [i.e., Germ Aversion (GA)] is composed of eight items to evaluate aversive affective responses to situations that connote a relatively high probability of transmission of pathogens. The subscale scores are computed as the mean of all items within a factor. Higher scores indicate greater perceived vulnerability to disease (Park and Justin, 2009). The 15 items showed an acceptable level of internal consistency, i.e., Cronbach's alpha = 0.82. For the seven items on the PI factor, Cronbach's alpha = 0.87. For the eight items on the GA factor, Cronbach's alpha = 0.74.

The State-Trait Anxiety Inventory (STAI; Spielberger and Sydeman, 1994) is a questionnaire that shows how strong the feelings of anxiety of a person are. It is composed of two scales, one to evaluate state anxiety [i.e., State Anxiety Inventory (SAI)] and the other to evaluate trait anxiety [i.e., Trait Anxiety Inventory (TAI)]. State anxiety refers to the state of mind (i.e., fear, nervousness, and discomfort) of a person at the time of a perceived threat and is considered temporary, while trait anxiety refers to the feelings of stress, worry, and discomfort that one can experience in typical situations of everyday life. Each scale asks 20 questions and is rated on a 4-point scale (i.e., 1 = not at all to 4 = very much so) (Grös et al., 2007). Scores range from 20 to 80, with higher scores correlating with greater anxiety. In this study, TAI showed a reliability score of 0.532, while SAI showed a reliability score of 0.588.

The Smartphone Addiction Scale Short Version for Adolescents and Young Adults (SAS-SV; De Pasquale et al., 2017) is a questionnaire to identify the risk of smartphone addiction. It distinguishes the high-risk group from the addicted group. The questionnaire includes 10 items describing daily life disturbance, positive anticipation, withdrawal, cyberspace-oriented relationships, overuse, and tolerance. For each item, participants expressed their opinion on a 6-point scale (i.e., 1 = strongly disagree to 6 = strongly agree). The scale is used to identify the different ranges for men and women. Men were addicted to scores above 31, with a high risk of addiction on scores between 22 and 31, and women were addicted to scores above 33, with a high risk of addiction on scores between 22 and 33 (Kwon et al., 2013; De Pasquale et al., 2017). In this study, SAS-SV showed high reliability (Cronbach's alpha = 0.79).

### Data Analysis

We performed a set of statistical analyses on our data (i.e., descriptive analyses, factors correlation, linear regression, and instrument reliability with Cronbach's alpha) using SPSS software version 26.

## Results

The results did not show significant gender differences in PVD, STAI (i.e., SAI and TAI versions), and SAS-SV scales calculated through the *t*-test of independent samples (Table 1). There were gender differences only in FCV-19S, and women showed more fear of COVID-19 than men. Nevertheless, the fear of COVID-19 (i.e., FCV-19S) did not reach an intensity such as to be defined as serious (i.e., fear score: 15.53) in the whole sample. Both men and women showed a high risk of smartphone addiction (i.e., score of males: 28.33 and score of females: 26.88) in SAS-SV (for the range of scores, see “Measures” section). University students showed moderate trait and state anxiety (i.e., a score of 51.60 in TAI and a score of 47.21 in SAI) in STAI (for the range of scores, see “Measures” section). In addition, students showed moderate perceived vulnerability to disease (i.e., a score of 51.51) in PVD (for the range of scores, see “Measures” section).

**Table 1:** Gender differences in FCV-19S, PVD, PI, GA, SAI, TAI, and SAS-SV measured using the *t*-test of independent samples.

	**Gender**	**Mean**	**SD**	***t***	***p-*value (*df* = 191)**	**Mean difference**	**SE difference**	**95% CI of the difference**	
								**Lower**	**Upper**
SAS-SV	M	28.33	9.00	1.13	0.257	1.44	1.27	−1.06	3.96
	F	26.88	8.66						
PI	M	19.76	5.55	−0.28	0.773	−0.23	0.80	−1.81	1.35
	F	20.00	5.56						
GA	M	31.39	5.65	−0.56	0.572	−0.49	0.87	−2.21	1.22
	F	31.88	6.32						
PVD	M	51.05	8.52	−0.64	0.519	−0.83	1.28	−3.36	1.70
	F	51.88	9.18						
FCV-19S	M	14.01	5.80	−3.12	0.002	−2.73	0.87	−4.46	−1.01
	F	16.75	6.24						
SAI	M	46.40	5.37	−1.76	0.080	−1.44	0.82	−3.06	0.17
	F	47.85	5.91						
TAI	M	51.01	6.04	−1.20	0.228	−1.07	0.88	−2.82	0.67
	F	52.08	6.20						

*M, Male; F, Female; FCV-19S, Fear of COVID-19 Scale; PVD, Perceived Vulnerability to Disease, PI, Perceived Infectability; GA, Germ Aversion; TAI, Trait Anxiety Inventory; SAI, State Anxiety Inventory; and SAS-SV, Smartphone Addiction Scale Short Version for Adolescents and Young Adults*.

The Pearson's correlations are shown in Table 2. GA, PI, and SAI were significantly correlated with fear of COVID-19 (i.e., FCV-19S) and trait anxiety (i.e., TAI). In contrast, they were not correlated with the risk of smartphone addiction (i.e., SAS-SV).

**Table 2:** Pearson's correlation (*r*) among FCV-19S, STAI, PVD, and SAS-SV.

	**PI**	**GA**	**PVD**	**FCV-19S**	**SAI**	**TAI**	**SAS-SV**
PI	1	0.172^*^	0.590^**^	0.148^*^	0.194^**^	0.239^**^	−0.008
GA	0.172^*^	1	0.804^**^	0.322^**^	0.316^**^	0.211^**^	0.036
PVD	0.590^**^	0.804^**^	1	0.400^**^	0.408^**^	0.352^**^	0.011
FCV-19S	0.148^*^	0.322^**^	0.400^**^	1	0.523^**^	0.223^**^	0.010
SAI	0.194^**^	0.316^**^	0.408^**^	0.523^**^	1	0.386^**^	−0.017
TAI	0.239^**^	0.211^**^	0.352^**^	0.223^**^	0.386^**^	1	0.004
SAS-SV	−0.008	0.036	0.011	0.010	−0.017	0.004	1

*FCV-19S, Fear of COVID-19 Scale; PVD, Perceived Vulnerability to Disease, PI, Perceived Infectability; GA, Germ Aversion; TAI, Trait Anxiety Inventory; SAI, State Anxiety Inventory; and SAS-SV, Smartphone Addiction Scale Short Version for Adolescents and Young Adults*.

* *Significance p < 0.05*.

** *Significance p < 0.01*.

Table 3 reports the results of the linear regression between the independent variables (i.e., FCV-19S and TAI) and dependent variables (i.e., SAI, SAS-SV, and PVD). The results showed that fear of COVID-19 and trait anxiety appear to be the predictors of state anxiety (i.e., SAI) and PVD, but not the predictors of risk of smartphone addiction (i.e., SAS-SV).

**Table 3:** Linear regression model between the independent variables (i.e., FCV-19S and TAI) and dependent variables (i.e., SAI, SAS-SV, and PVD) with 95% bias-corrected and CIs.

	**Standardized coefficients**	***t***	***p*-value**	**95% CI for Regression coefficients (B)**	
	**β**			**Lower bound**	**Upper bound**
	**SAI**				
Constant		9.55	<0.001	21.43	32.59
FCV-19S	0.46	7.68	<0.001	0.31	0.53
TAI	0.28	4.74	<0.001	0.15	0.37
	**SAS-SV**				
Constant		5.02	<0.001	16.51	37.89
FCV-19S	0.00	0.12	<0.001	−0.19	0.22
TAI	0.00	0.02	<0.001	−0.20	0.21
	**PVD**				
Constant		4.88	<0.001	13.91	32.76
FCV-19S	0.33	5.20	<0.001	0.30	0.67
TAI	0.27	4.25	<0.001	0.21	0.58

*t, t-test; p, p-value; FCV-19S, Fear of COVID-19 Scale; PVD, Perceived Vulnerability to Disease, PI, Perceived Infectability; GA, Germ Aversion; TAI, Trait Anxiety Inventory; SAI, State Anxiety Inventory; and SAS-SV, Smartphone Addiction Scale Short Version for Adolescents and Young Adults*.

## Discussion and Conclusions

The data from this study did not show significant differences regarding gender in the various assessment tests performed, except in the fear of COVID-19, in which women showed greater fear. Both male and female students showed a significant score for a high risk of smartphone addiction at SAS-SV, while the fear of COVID-19 did not reach a level to be defined as severe. Both state and trait anxieties showed a level of moderate severity, as well as a moderate perception of vulnerability to infections. Although the fear of COVID-19 cannot be defined as serious in the examined students, the correlations showed how, as the fear of the pandemic increased, both the aversion to germs and the perception of infections increased.

Furthermore, the same variables (i.e., FCV-19S and PVD) were also positively correlated with trait anxiety (i.e., TAI). We hypothesized that the trait anxiety highlighted in the examined subjects could have encouraged preventive and control behaviors toward the fear of contagion. It is significant to note that, although the use of smartphones can be defined as having a high risk of addiction, no significant correlations emerged between the latter and the other dimensions examined. Among other things, the PSU did not appear to depend on the fear of COVID-19.

This seems to be in line with some other studies carried out on student samples during the COVID-19 pandemic. For example, the study by David and Roberts (2021) conducted on 400 college students found that smartphone use mitigated the negative impact of social distancing on social connection and well-being. Therefore, contrary to a popular opinion regarding the negative influence of smartphone use on well-being, its increased use during the pandemic could foster social connection and well-being.

Moreover, some other studies on young adults showed that, although depression and social anxiety were the determinants of the risk for a greater PSU, particular categories of technological applications were positively correlated with psychophysical well-being (De Pasquale et al., 2020; Pera, 2020).

Another significant finding that emerged from this study concerns the predictive value of fear of COVID-19 and trait anxiety on the perception of vulnerability to infections. Therefore, knowing in advance the psychological traits of the subjects, it is possible to intervene in time, preventing the negative influences of a particularly stressful event from becoming serious.

This study has some limitations. In particular, the study reveals some methodological shortcomings, as the perception scales of vulnerability to disease have not typically been designed for clinical diagnostic purposes, diminishing their suitability for clinical research. Further study needs to be conducted to explore further phenomena that could be predicted by the constructs evaluated by the PVD. Furthermore, the sample of subjects examined is relatively small and concerns only a portion of University students in the city of Catania, even if the results of the study are quite consistent with the study carried out so far on University students in other countries during the COVID-19 pandemic. Another important limitation to consider concerns the fact that the state of the emotional well-being of these subjects is not known before the pandemic period, and therefore, we do not know for sure if the moderate state of anxiety found depends only on the effects of the pandemic or also on other variables. It is not possible to exclude states of anxiety and the presence of a certain degree of perception of vulnerability to infections preceding the pandemic due to the absence of evaluation.

The data highlighted the presence of the perception of vulnerability to infections in subjects with moderate anxiety, both state and trait, to be associated with the fear of the COVID-19 pandemic. It is hoped that a large part of the population will soon be vaccinated, including University students, and therefore, it would be desirable to carry out further assessments in the post-vaccine phase to highlight any differences in the state of anxiety and the perception of vulnerability to infections. The possible positive role of the use of smartphones in maintaining social contacts should also be emphasized.

## Data Availability Statement

The raw data supporting the conclusions of this article will be made available by the authors, without undue reservation.

## Ethics Statement

The studies involving human participants were reviewed and approved by Italian Association of Psychology. The patients/participants provided their written informed consent to participate in this study.

## Author Contributions

CD: conceptualization, methodology, data curation, writing—original draft preparation, writing—review and editing, supervision, and funding acquisition. MLP: methodology, investigation, and writing—original draft preparation. FS: methodology, validation, formal analysis, investigation, and writing—original draft preparation. ZH: writing—review and editing, visualization, and supervision. All authors contributed to the article and approved the submitted version.

## Conflict of Interest

The authors declare that the research was conducted in the absence of any commercial or financial relationships that could be construed as a potential conflict of interest.
